# Immune infiltration and PD-L1 expression in the tumor microenvironment are prognostic in osteosarcoma

**DOI:** 10.1038/srep30093

**Published:** 2016-07-26

**Authors:** Pratistha Koirala, Michael E. Roth, Jonathan Gill, Sajida Piperdi, Jordan M. Chinai, David S. Geller, Bang H. Hoang, Amy Park, Michael A. Fremed, Xingxing Zang, Richard Gorlick

**Affiliations:** 1 Department of Molecular Pharmacology, Albert Einstein College of Medicine, Bronx, NY, USA; 2 Division of Pediatric Hematology, Oncology, Marrow & Blood Cell Transplantation, Children’s Hospital at Montefiore, Albert Einstein College of Medicine, Bronx, NY, USA; 3 Department of Microbiology and Immunology, Albert Einstein College of Medicine, Bronx, NY, USA; 4 Department of Orthopedic Surgery, Montefiore Medical Center, Albert Einstein College, Bronx, NY, USA; 5 New York–Presbyterian Morgan Stanley Children’s Hospital, Columbia University Medical Center, New York, New York, USA

## Abstract

Osteosarcoma patient survival has remained stagnant for 30 years. Novel therapeutic approaches are needed to improve outcomes. We examined the expression of Programmed Death Ligand 1 (PD-L1) and defined the tumor immune microenvironment to assess the prognostic utility in osteosarcoma. PD-L1 expression in osteosarcoma was examined in two patient cohorts using immunohistochemistry (IHC) (n = 48, n = 59) and expression was validated using quantitative real time PCR (n = 21) and western blotting (n = 9). IHC was used to determine the presence of tumor infiltrating lymphocytes and antigen-presenting cells (APCs) in the tumor. Expression of PD-L1 was correlated with immune cell infiltration and event-free-survival (EFS). The 25% of primary osteosarcoma tumors that express PD-L1 were more likely to contain cells that express PD-1 than PD-L1 negative tumors (91.7% vs 47.2%, p = 0.002). Expression of PD-L1 was significantly associated with the presence of T cells, dendritic cells, and natural killer cells. Although all immune cell types examined were present in osteosarcoma samples, only infiltration by dendritic cells (28.3% *vs.* 83.9%, p = 0.001) and macrophages (45.5% *vs.* 84.4%, p = 0.031) were associated with worse five-year-EFS. PD-L1 expression was significantly associated with poorer five-year-EFS (25.0%. *vs.* 69.4%, p = 0.014). Further studies in osteosarcoma are needed to determine if targeting the PD-L1:PD-1 axis improves survival.

Osteosarcoma is the most common primary malignant bone tumor in children and young adults^1^,^2^,^3^. Although there have been great advances in the treatment of osteosarcoma, survival rates have remained stagnant over the past three decades. With the introduction of multimodal therapy, including surgery and chemotherapy, 5-year event-free survival (EFS) has remained less than 70%, which has remained unchanged over the past few decades^4^,^5^. Patients with metastatic or relapsed disease have particularly dismal outcomes, with a less than 30% 5-year EFS^6^,^7^,^8^,^9^,^10^. There is an urgent need for new therapeutic approaches to improve outcomes for these patients.

In recent years, numerous studies have sought to define the interplay between the immune system and tumor, with the potential to target this interaction for therapeutic intervention. In many malignancies, both innate and adaptive immune cells play a role in the tumor microenvironment, with communication between natural killer (NK) cells, antigen presenting cells (APCs) such as macrophages and dendritic cells (DC), and lymphocytes allowing for effective tumor control^11^. Aberrations in the function of immune cells can lead to ineffective tumor surveillance and tumor elimination. Abnormal immune function can also contribute to enhanced tumor growth by increasing local inflammation, creating an environment conducive to tumor growth^12^,^13^,^14^.

Immune cells are initially attracted to tumor cells by the presence of tumor specific antigens. Cancers with higher mutational loads present greater numbers of tumor specific neoantigens and are frequently associated with robust immune infiltration^11^,^15^. Osteosarcoma demonstrates significant genetic complexity, with the majority of tumors displaying loss of both p53 and RB^16^,^17^,^18^. In addition, 33% of primary osteosarcoma shows evidence of chromothripsis, or chromosome shattering^19^,^20^, and over 50% exhibit kataegis, or localized areas of hypermutation^18^,^21^. The high mutational load in osteosarcoma, along with the regular interaction between immune cells and bone cells in normal tissue, suggests that osteosarcoma may be an immunogenic tumor and evasion of the immune response may be an important component of its pathogenesis. Although previous studies have suggested a potential role for immune cells in osteosarcoma, to date no study has provided a comprehensive picture of the immune microenvironment^22^,^23^.

Tumors escape immunosurveillance by expressing immune checkpoints, such as programmed death ligand-1 (PD-L1)^24^,^25^. The interaction between PD-L1, expressed on tumor cells and its receptor, programmed death-1 (PD-1), expressed on immune cells, leads to immune cell apoptosis, anergy, and tolerance. Recent studies have demonstrated that cancers like melanoma, despite being highly immunogenic tumors, are not effectively cleared, partially due to their ability to express PD-L1^26^. A number of studies have already demonstrated that blockade of the PD-L1:PD-1 interaction in patients with Hodgkin lymphoma, non-small-cell lung cancer, and melanoma leads to improved outcomes^27^,^28^, and anti-PD-1 antibodies have been FDA approved for the treatment of melanoma^29^,^30^,^31^. In addition, studies have reported that tumor response to PD-L1 or PD-1 inhibition is directly related to the level of PD-L1 expression and lymphocytic infiltration of the tumor^32^,^33^,^34^,^35^.

Previous studies have begun to examine the role of PD-L1 in osteosarcoma. Utilizing quantitative real-time PCR (qRT-PCR), PD-L1 mRNA expression was associated with lymphocyte infiltration^23^. Subsequent studies, utilizing immunofluorescence (IF), reported PD-L1 was not expressed in primary specimens and was only expressed in metastatic tissue^22^. Further studies examining PD-L1 blockade in a mouse model of osteosarcoma showed initial regression of the tumor followed by growth of PD-L1 antibody resistant clones. In response to treatment with anti-PD-L1 antibody, tumor cells downregulated PD-L1 and upregulated CD80 and CD86. In addition, neighboring T-cells exhibited decreased expression of PD-1 and increased expression of CTLA4, suggesting that alternate immune checkpoints may play a role is osteosarcoma resistance to PD-L1 blockade^36^. Combination therapy targeting PD-L1 and CTLA-4 showed control of osteosarcoma growth in the majority of tumors in a mouse model^22^,^36^. In this study, we determined the potential prognostic and therapeutic utility of PD-L1 by examining its expression and characterizing the immune microenvironment of human osteosarcoma.

## Results

### PD-L1 is expressed in primary osteosarcoma tissue

**Figure 1:**
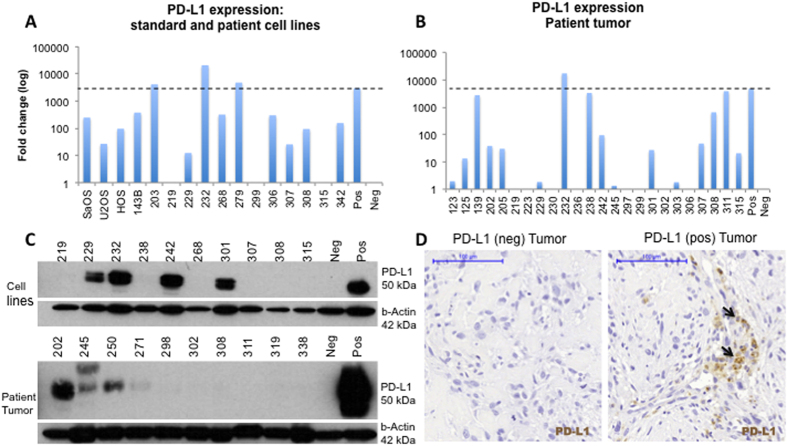
PD-L1 is expressed in some primary osteosarcoma tumors. (**A**) PD-L1 mRNA expression in cell lines derived from primary osteosarcoma tumors. The PD-L1 expressing breast cancer cell line MDA-MB-231 was used as the positive control. (**B**) PD-L1 mRNA expression in osteosarcoma tumor specimens. Dashed lines represent the level of PD-L1 expression in the positive control. (**C**) Western blot validation confirms that PD-L1 is expressed in primary osteosarcoma tumors (30%) (**D**) IHC on selected osteosarcoma tumor specimens show areas of heterogeneous PD-L1 expression. Scale bar represents 100 μm. Neg = negative, pos = positive.

PD-L1 expression was quantified in osteosarcoma patient and standard cell lines by qRT-PCR. Three quarters of patient derived cell lines, all the osteosarcoma standard cell lines (HOS, HOS-143b, SaOS, and U2OS), and two thirds of tumor specimens demonstrated expression of PD-L1 mRNA greater than the negative MCF7 control cell line; however, only a small subset expressed PD-L1 at the same level as the positive control, breast cancer cell line MDA-MB-231. Specifically, three of the sixteen cell lines (203, 232, and 279), 19% (Fig. 1A), and four of the 24 tumors (311, 139, 232, 238), 17%, were highly positive for PD-L1 mRNA, with expression levels near or exceeding the positive control (Fig. 1B).

PD-L1 protein expression was validated with western blotting: 3 of 10 patient samples (30.0%) and 4 of 10 osteosarcoma patient derived cell lines (40%) demonstrated detectable levels of PD-L1 protein (Fig. 1C). mRNA and protein expression did not correlate as some tumors that had mRNA expression as detected by qPCR but no detectable protein expression, specifically cell lines 307 and 308 and tumors 308 and 311.

PD-L1 protein expression was subsequently examined using IHC (Fig. 1D) on an osteosarcoma TMA containing 54 patient samples (Table S1). PD-L1 expression was quantified as the percentage of positive tumors cells on the slide compared to the tumor volume. Seven percent of all specimens on the TMA stained positive for PD-L1, with the majority of positive tumors demonstrating less than 25% staining. No significant differences in PD-L1 expression were seen between primary specimens and metastatic specimens (6.8% vs 15.4%, p = 0.24). Due to the low percentage of PD-L1 positive primary tumors detected by IHC compared to qPCR and western blotting, PD-L1 staining was performed on a second cohort of samples using whole slides, which included increased area of osteosarcoma tissue per sample. In the whole slide cohort, 12 patients (25.0%) had positive PD-L1 expression, of which eight had associated clinical data (Table 1). All of the positive samples demonstrated <25% PD-L1 staining.

### PD-L1 positive tumors are associated with the presence of tumor infiltrating immune cells in the tumor microenvironment

**Figure 2:**
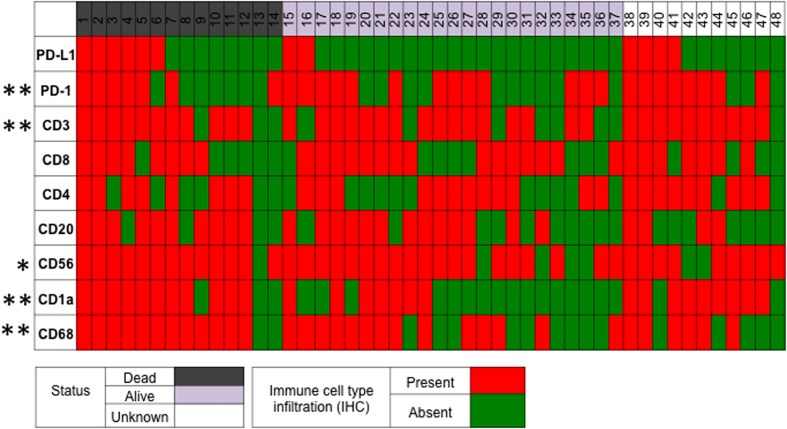
Expression of the immune checkpoint, PD-L1, is associated with the presence of tumor infiltrating lymphocytes and antigen presenting cells. Each individual column represents a unique patient; of the 28 patients included in the study, 37 patients had known clinical outcomes, and 29 patients had both clinical outcome data and as well as demographic data available. Rows represent either PD-L1 expression or tumor infiltration by an immune cell type, as determined by immunohistochemistry. PD-L1 expression was significantly associated with infiltration by PD-1+ immune cells, CD3+ T cells, CD56+ natural killer cells, CD68+ cells, and CD1a+ dendritic cells. Presence of immune cells or positive PD-L1 expression was defined as IHC staining of >1% the tumor volume. * p < 0.05, **p < 0.01.

The presence of six groups of tumor infiltrating immune cells was examined by IHC in whole slides (Figures S1–S6). Presence of the immune cell was classified as a tumor that had greater than 1% of the tumor volume consisting of positively stained cells. Tumor infiltrating lymphocytes (TILs) examined included multiple T-lymphocyte subsets as well as B-lymphocytes. Additionally, slides were stained for the presence of macrophages, DCs, and PD-1 positive cells. Ninety eight percent of all osteosarcoma tumors examined demonstrated infiltration by at least one immune cell type, including 18.8% that demonstrated infiltration by all immune cell types assessed (Fig. 2).

PD-L1 positive tumors were associated with infiltration by multiple immune cells types as compared to PD-L1 negative tumors, which demonstrated infiltration by fewer immune cells types. Tumors expressing PD-L1 were significantly more likely to demonstrate infiltration by all immune cell types compared with PD-L1 negative tumors (50.0% vs 5.6%, p = 0.002). Tumors expressing PD-L1 compared to those that were PD-L1 negative were more likely to demonstrate infiltration by CD3+ T-cells (91.7% vs 55.6%, p = 0.002), CD56+ NK cells (100% vs. 66.7%, p = 0.02), CD68+ macrophages (91.7% vs 50%, p = 0.02), and CD1a+ DCs (83.3% vs 30.6%, p = 0.002). In addition, immune cells were significantly more likely to express PD-1 in PD-L1 positive tumors compared with PD-L1 negative tumors (91.7% vs 47.2%, p = 0.002) (Table S2).

### Expression of PD-L1 is consistent within the tumor mass

**Figure 3:**
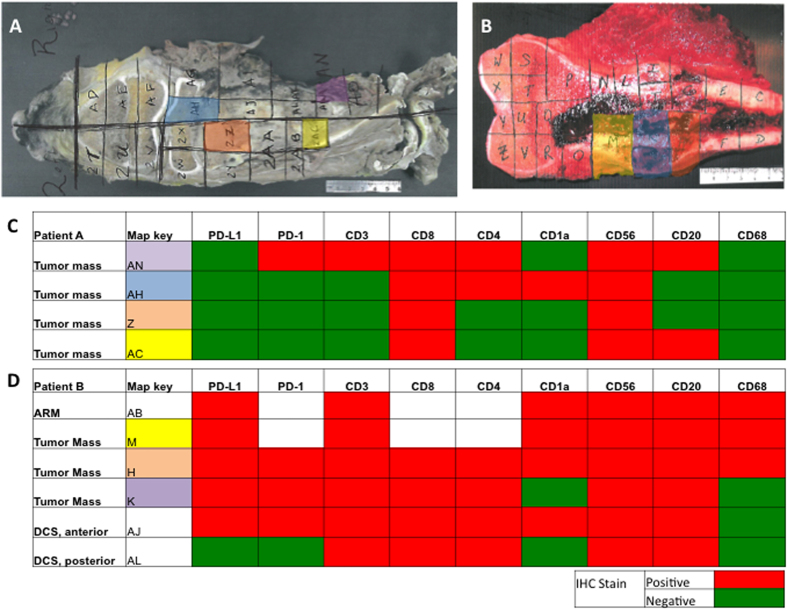
PD-L1 expression is consistent throughout the tumor mass. (**A**,**C**) All four slides from the tumor mass of patient A did not demonstrate PD-L1 staining. Few immune cells are present in this tumor map. (**B**,**D**) All of the slides from the tumor mass of patient B stained PD-L1 positive with the exception of one section outside the tumor mass (not shown). Numerous immune cells are present throughout all sections of this tumor map. ARM = anterior resection margin, DCS = distal cross section.

IHC indicated that PD-L1 expression and immune cell infiltration is heterogeneous within the whole slide samples examined. Two osteosarcoma tumor maps, containing slides from multiple areas of tumor, were examined in order to assess the consistency of PD-L1 expression and tumor infiltrating immune cells across whole tumors. Four slides were selected from the map for patient A, containing tissue from four locations across the tumor mass (Fig. 3A). Six slides were selected from the map for patient B, containing tissue from three locations across the tumor mass and three locations containing the resection margin (Fig. 3B).

Patient A’s tumor was PD-L1 negative across all sections examined (Fig. 3C) and patient B’s tumor was PD-L1 positive in five out of six sections, with only one region located outside of the tumor mass demonstrating negative PD-L expression (Fig. 3D). In both tumors, presence of specific tumor infiltrating cells varied only slightly across multiple sections of the tumor, with the exception of CD56+ NK cells and CD8+ T-regulatory cells, which were universally present. Overall, the PD-L1 positive tumor contained more tumor infiltrating immune cells types than did the PD-L1 negative tumor.

### PD-L1 expression or APC infiltration is associated with event free survival

**Figure 4:**
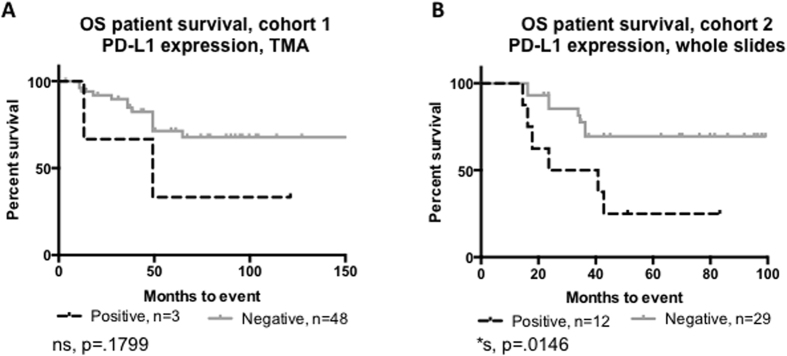
PD-L1 expression is prognostic in osteosarcoma. (**A**) PD-L1 expression in the TMA trends with poorer EFS. (**B**) PD-L1 expression in the whole slide specimens is significantly associated with poorer EFS.

In order to assess the prognostic relevance of PD-L1 and tumor infiltrating immune cells in osteosarcoma, Kaplan Meier curves were generated. EFS, with an event being defined as relapse or death, was calculated based on presence or absence of PD-L1 protein and presence of specific immune cells as determined by IHC on both TMA and whole slide samples. PD-L1 expression was associated with significantly poorer five-year EFS in the whole slide samples (25% *vs.* 69.4%, p = 0.01). In contrast, although the TMA cohort showed a trend for poorer survival with PD-L1 expression (33.3% *vs.* 71.4%, p = 0.18), this result did not achieve statistical significance (Fig. 4A,B).

**Figure 5:**
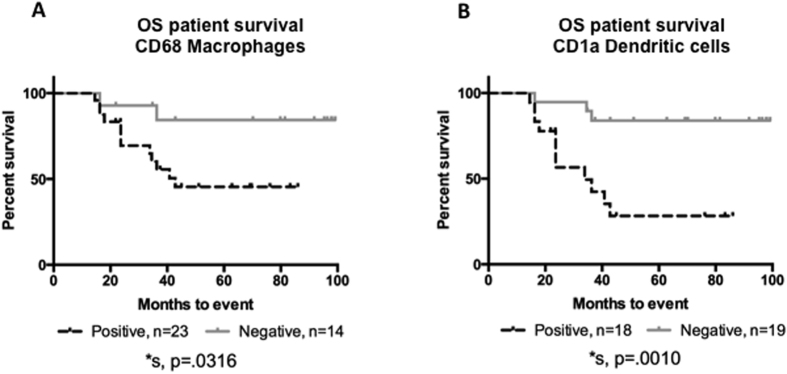
APC infiltration is prognostic in osteosarcoma. Presence of APCs, specifically (**A**) CD68 positive macrophages or (**B**) CD1a positive dendritic cells are significantly associated with poorer EFS in osteosarcoma. Over 50% of osteosarcoma samples have APCs present.

In the whole slide specimens, tumor infiltration by APCs was associated with poorer 5 yr-EFS, including CD1a+ DCs (28.3% *vs.* 83.9%, p = 0.001) and CD68+ macrophages (45.5% *vs.* 84.4%, p = 0.032) (Fig. 5). Infiltration by the other immune cells assessed was not associated with differences in the 5-year EFS. (Figures S1–S6).

## Discussion

In this study we demonstrated that PD-L1 was expressed in up to 25% of osteosarcoma samples, as determined by IHC using whole slide tumor sections. It is noteworthy that only 6.8% of osteosarcoma tumors, examined using a TMA containing 1 mm cores, were PD-L1 positive. It seems likely that this discrepancy between whole slides and the TMA is a result of the heterogeneity of PD-L1 expression in osteosarcoma—although PD-L1 positive cells were robustly stained, they made up a small fraction of cells examined on whole slide tumor sections. It is also possible that creation of the TMA, which is created to maximize areas of viable tumors, may bias against areas that are PD-L1 positive. These results suggest that IHC using a 1mm TMA may underestimate the number of PD-L1 positive osteosarcoma tumors.

A previous study, using a 2^ΔCT^ method, found that the 84.2% of primary osteosarcoma tumors express detectable PD-L1 mRNA^23^. PD-L1 expression appears to be conserved across a number of solid tumors and hematologic malignancies. PD-L1 protein expression in a variety of cancers, such as melanoma (40–100%), non-small cell lung cancer (35–95%), and lymphomas (17–94%) is comparable to the range detected in osteosarcoma^37^. A study validating the prognostic and therapeutic value of a new anti-PD-L1 antibody defined PD-L1 positivity as >5% of tumor-infiltrating cells or tumor cells staining for PD-L1^34^. Although we used a lower threshold of PD-L1 positivity, the previous study suggests that a low level of PD-L1 expression may have clinically meaningful impact.

Our results validated pervious PD-L1 mRNA expression reported in osteosarcoma^23^, but inclusion of a positive control allowed us to have a higher and more stringent cutoff for what we considered a truly positive tumor. Regardless, we found that the mRNA levels do not consistently correlate with protein expression, as determined by both IHC and western blotting. A second group was able to detect PD-L1 expression using immunofluorescence in approximately 75% of metastatic, but not primary, tissues^22^. This may reflect the increased propensity of metastatic osteosarcoma to express PD-L1 but it may also be due to limitations in the antibody available at that time. In order to address this issue we tested multiple PD-L1 antibodies on known positive and negative controls and subsequently optimized the chosen antibody in osteosarcoma (Table S3). Because previous studies have shown that PD-L1 protein expression by IHC is a predictor of response to both anti-PD-L1 and anti-PD-1 therapy in a variety of cancers^32^,^33^,^34^,^35^, it may be important to correctly identify patients who may benefit from this potential intervention.

In this study, we have shown that PD-L1 expression in primary tumors may be a prognostic marker for poorer survival. In addition to identifying PD-L1 as a potential prognostic marker that may be present in up to 25% of osteosarcoma cases, we demonstrated that PD-L1 positive osteosarcoma tumors have higher numbers of TILs and APCs than their PD-L1 negative counterparts. Although infiltration by TILs is significantly higher in PD-L1 positive tumors and their presence trends with worsened survival, there is no significant association between TILs and patient survival. On the other hand, presence of either of the APCs examined, was significantly associated with worsened survival.

Tumor associated macrophages (TAMs) are present in multiple solid tumors, including breast, gastric, and ovarian cancers^38^. Multiple studies have shown that TAMs play an important role in breast cancer pathogenesis by promoting angiogenesis, tumor cell invasion, and migration^39^,^40^,^41^,^42^. Unlike the presence of TAMs in other solid tumors, the presence of CD14 positive immune cells, which include macrophages, monocytes, neutrophils, and dendritic cells^43^, was previously found to be associated with decreased metastasis but increased angiogenesis in osteosarcoma^44^. The presence of M1 macrophages, which are pro-inflammatory but not pro-tumor, has been associated with better outcomes in osteosarcoma. Treatment of osteosarcoma with muramyl-tripeptide, which appears to activate monocytes to become M1 macrophages, causing a cytokine cascade^45^, leads to osteosarcoma cell death *in vitro*^46^.

Although previous studies may suggest that the opposite may be the case, we found that the presence of CD68+ TAMs in primary tissue may be significantly associated with worse survival in osteosarcoma. In previous studies, by capturing both monocytes and mature (M1 and M2) macrophages with CD14 staining, the role of mature macrophages may have been confounded. In our current study CD68 staining captures both pro-inflammatory M1 and pro-tumor M2 macrophages. In order to clarify the role of macrophages in osteosarcoma, future studies should correlate the presence of CD68+/CD80+ M1 and CD68+/CD163+ M2 macrophages to osteosarcoma survival.

It is also possible that osteoclasts, a subset of CD68+ bone specific macrophages, may impact osteosarcoma patient survival. Presence of osteoclasts in osteosarcomas have been linked to conflicting outcomes in past studies^47^. Although a recent study shows that pulmonary osteosarcoma metastases have fewer osteoclasts^48^, earlier work has shown that patients with aggressive osteosarcomas often have increased osteoclast activity^49^ and that inhibition of osteoclast function via a RANK-ligand antagonist leads to favorable outcomes in mice^50^.

Presence of a second APC, CD1a+ DCs, is also associated with poor survival in osteosarcoma. With the recent advent of DC vaccines as a cancer treatment, multiple groups have shown that DC vaccines have varying levels of effectiveness in animal models of osteosarcoma^51^,^52^,^53^,^54^. It has been previously shown that human osteosarcoma cells in culture inhibit the ability of DC to present antigens^55^. Furthermore, human bone does not contain lymphatic vessels, implying that DCs cannot function in bone as they do in other tissues^56^. However, osteosarcomas frequently have a soft tissue component which may contain lymphatic vessels. The relevance of lymphatic vessels in osteosarcoma is not clear, however, the primary mode of metastasis in osteosarcoma is via hematogenous spread, with fewer than 4% of patients displaying lymph node metastasis^57^,^58^. It seems that despite showing the potential of vaccines in animal models, DCs may be unable to function properly in human osteosarcoma.

A major limitation of this study is the small number of samples available in this single institution analysis of the tumor immune microenvironment in osteosarcoma. Our sample size was further limited by the lack of associated clinical data for a subset of tumors. Although the non-significant correlative outcome results in the TMA may be explained by the limited sample assessed in each core, and the limited sample number, these represent two different patient cohorts and we cannot rule out the possibility that the prognostic correlation is purely a random occurrence in a small patient population. Further studies are necessary to validate that these results can be recapitulated in a larger multi-institutional cohort.

In conclusion, we have shown that tumor infiltration by either CD68+ TAMs or CD1a+ DCs may be associated with poorer survival in osteosarcoma. Furthermore, PD-L1 is expressed in a subset of primary osteosarcoma samples at the time of biopsy, and it may be prognostic for increased mortality. PD-L1 expression is associated with TILs and APCs, including PD-1 positive immune cells. Due to its expression in a significant number of osteosarcoma tumors and an abundant presence of immune cells, the PD-L1:PD-1 axis may be a promising target for immunotherapy in osteosarcoma, particularly given osteosarcoma known genetic complexity. Further evaluation of PD-L1 protein expression is warranted in a larger cohort of patients with osteosarcoma. Currently a phase II study of pembrolizumab (anti-PD-1 antibody) in patients with advanced sarcomas, including osteosarcomas, is ongoing, the results of which may be informative.

## Materials and Methods

### Cell Cunlture and Human Materials

Both osteosarcoma standard cells lines (HOS, HOS-143b, SaOS, and U2OS) and isolated cells were cultured in Eagle minimum essential medium supplemented with 20% fetal bovine serum (FBS) and antibiotics. PD-L1 negative MCF7 breast cancer cells, PD-L1 positive MDA-MB-231breast cancer cells, and PD-L1 negative 3T3 cells were cultured in Dulbecco’s modified Eagle’s medium supplemented with 10% FBS. Primary osteosarcoma cultures were generated using standard collagenase disaggregation of surgical specimens from seven osteosarcoma patients, as has been described previously^59^. All cell lines were grown in 5% CO_2_ humidified atmosphere at 37 °C.

Tumors collected from two cohorts of osteosarcoma patients were used to construct a TMA or whole slides, as previously described^60^,^61^. Tissue was decalcified overnight, formalin-fixed, and paraffin embedded. Four to five-micron thick sections were cut from representative areas of the tumor and used for immunohistochemistry (IHC). In total, the TMA contained cores from 62 samples representing 51 unique patients, of which 54 cores were usable (Table S1). The cores that detached from the slide during processing were deemed unusable. Slides with an area of 50–135 cm^2^ were constructed using tumors collected from a second cohort of patients (Table 1), and were used to validate the TMA results. This second cohort included 48 total patients, of which 37 patients had associated clinical outcomes and 29 had clinical outcomes and additional demographic information.

The Ethics Committees and Institutional Review Boards at Montefiore Hospital, Memorial Sloan Kettering Cancer Center, and the Center for Cancer Research approved the study. Written informed consent was obtained from the patients and their parents/guardians prior to tissue collection. All procedures were conducted in accordance with guidelines provided by the Ethics Committees and Institutional Review Boards.

### Protein extraction and Western Blotting

Protein was extracted from 15 frozen patient tumors using RIPA buffer (ThermoScientific). The tumors were collected in accordance with an IRB approved research protocol with patient/guardian informed consent as described previously^62^. Proteins were denatured in a reducing sample buffer and run on a 12% tris gel using tris-glycine buffer. Proteins were transferred to a nitrocellulose membrane, incubated with 5% milk block, probed with antibody for PD-L1 and subsequently with either a goat anti-rabbit HRP conjugated secondary antibody (Cell Signaling) diluted in 5% bovine serum albumin. Horse radish perodixase conjugated secondary antibodies were detected using autoradiography using chemoluminescence (Amersham Enhanced Chemiluminescence Prime Western Blotting Detection Reagent). 3T3 cells and MDA-MB-231 cells were used as a negative and positive control for PD-L1 expression, respectively.

### RNA extraction and quantitative PCR (qPCR)

RNA was extracted from flash frozen osteosarcoma tumor samples from 15 patients using Trizol reagent (Invitrogen) and converted to cDNA using Superscript II transcriptase (Life Technologies). qPCR was performed using commercially available TaqMan primers for PD-L1 (Life Technologies, Hs01125301_m1). Relative gene expression was normalized to an internal control, GAPDH, and calculated using the 2^−ΔΔCT^ method. MCF7 and MDA-MB-231 cell lines were used as a negative and positive control for PD-L1 mRNA expression, respectively. Due to lack of detectable amplification of PD-L1 in the negative control, the CT value for MCF7 was set to 40 for calculations.

### Tumor microarray (TMA) construction

Tumors, collected from osteosarcoma patients were used to construct a TMA, as has been described previously^60^,^61^. The TMA contains cores from osteosarcoma tumors collected at the time of biopsy and definitive surgery, as well as from metastatic tissues. Tumor specimens obtained at the time of biopsy or definitive surgery were all obtained from the patients’ primary site of disease. A pathologist reviewed the tumors in order to confirm the diagnosis of osteosarcoma. The TMA was constructed using 1-millimeter (mm) cores that were acquired from the formalin-fixed paraffin embedded tissue block from each individual tumor. Five-micron thick sections were cut and used for subsequent analysis^63^.

### Tumor Maps

Multiple slides were selected from tumor maps that were previously generated for clinical purposes using osteosarcoma specimens obtained with parental consent from two patients enrolled in an IRB-approved bio-banking protocol at Montefiore Medical Center. Samples were analyzed from multiple locations within the tumor mass and surrounding tissue at the time of definitive surgery following neoadjuvant chemotherapy. A set of slides was generated for each patient and this was related to the specimen map, which provided each slide’s location within the tumor.

### Immunohistochemistry

Immune staining was optimized for proper antigen retrieval and antibody concentrations using immune tissue for positive controls and brain tissue for negative controls. Osteosarcoma tumor slides were baked at 60 °C for one hour and antigen retrieval was performed using either tri-citrate buffer, pH 6.0 or tris-EDTA buffer pH 8.0 at 100 °C for 20 minutes. Slides were de-paraffinized using xylene, rehydrated along an ethanol gradient, and blocked using dual endogenous enzyme-blocking reagent (Dako) and a subsequent normal goat serum block (Santa Cruz). Slides were incubated overnight at 4 °C in primary antibody followed by a one hour room-temperature incubation with secondary antibody. Antibody concentrations and information can be found in Supplementary Table 1. Antigen expression was detected by addition of VECTASTAIN Elite ABC Kit (Vector Laboratories) and DAB (3,3-diaminobenzidine) HRP substrate (Vector Laboratories). Slides were counterstained with hematoxylin (Harris). Human placenta was used as positive control of PD-L1 expression. A full list of antibodies and their positive controls for all antibodies can be found in Supplementary Table 1.

### Quantification of IHC

Slides were scanned and graded by three independent reviewers without previous knowledge of patient outcomes or demographics. Slides were graded with a quartile score based on the percentage of positive staining cells present in the core/slide (0 = 0% staining, 1 = <25%, 2 = 25–50%, 3 = 50–75%, and 4 = >75%). If scores between the three graders were discordant the average, rounded to the nearest whole integer, was used. A tumor with a score of 1 or higher, representing ≥1% staining, was considered positive for PD-L1 expression or immune cell infiltration. Images of IHC staining were obtained at 20× magnification using the Pannoramic Scanner and captured using Pannoramic Viewer (3DHISTECH Ltd).

### Statistical analysis

Patient demographic data from each cohort was reported as frequencies for categorical variables and means with standard deviations for continuous variables. The association between the expression of PD-L1 and the presence of immune cells was assessed using Fisher’s exact test or chi square analysis. Kaplan Meier curves were generated assessing the association between EFS and PD-L1 expression or immune cell infiltration, and the Log-rank (Mantel-Cox) test was utilized (GraphPad Prism software). Survival curves were generated using expression thresholds of negative, defined as no staining (grade 0) and positive defined as at least 1% staining (grade 1, 2, 3, or 4).

## Additional Information

**How to cite this article**: Koirala, P. *et al*. Immune infiltration and PD-L1 expression in the tumor microenvironment are prognostic in osteosarcoma. *Sci. Rep.* **6**, 30093; doi: 10.1038/srep30093 (2016).

## Supplementary Material

Supplementary Information

## Figures and Tables

**Table 1:** Patient demographic information.

	Osteosarcoma patients (n = 33)
Median age (SD)	16 (5)
Huvos Response	
I/II standard	22
III/IV poor	11
Metastasis	
Present	4
Absent	29

## References

[b1] Damron T. A., Ward W. G. & Stewart A. Osteosarcoma, chondrosarcoma, and Ewing’s sarcoma: National Cancer Data Base Report. Clin Orthop Relat Res. 459, 40–47, 10.1097/BLO.0b013e318059b8c9 (2007). 17414166

[b2] Sweetnam R. Osteosarcoma. Br J Hosp Med. 28(112), 116–121 (1982). 6957252

[b3] Ottaviani G. & Jaffe N. The epidemiology of osteosarcoma. Cancer Treat Res. 152, 3–13, 10.1007/978-1-4419-0284-9_1 (2009). 20213383

[b4] Carrle D. & Bielack S. Osteosarcoma lung metastases detection and principles of multimodal therapy. Cancer Treat Res. 152, 165–184, 10.1007/978-1-4419-0284-9_8 (2009). 20213390

[b5] Harting M. T. & Blakely M. L. Management of osteosarcoma pulmonary metastases. Semin Pediatr Surg. 15, 25–29, 10.1053/j.sempedsurg.2005.11.005 (2006). 16458843

[b6] Link M. P. . The effect of adjuvant chemotherapy on relapse-free survival in patients with osteosarcoma of the extremity. N Engl J Med 314, 1600–1606, 10.1056/NEJM198606193142502 (1986). 3520317

[b7] Meyers P. A. . Addition of pamidronate to chemotherapy for the treatment of osteosarcoma. Cancer 117, 1736–1744, 10.1002/cncr.25744 (2011). 21472721 PMC3059356

[b8] Mirabello L., Troisi R. J. & Savage S. A. Osteosarcoma incidence and survival rates from 1973 to 2004: data from the Surveillance, Epidemiology, and End Results Program. Cancer 115, 1531–1543, 10.1002/cncr.24121 (2009). 19197972 PMC2813207

[b9] Bielack S. S. . Second and subsequent recurrences of osteosarcoma: presentation, treatment, and outcomes of 249 consecutive cooperative osteosarcoma study group patients. J Clin Oncol. 27, 557–565, 10.1200/JCO.2008.16.2305 (2009). 19075282

[b10] Hawkins D. S. & Arndt C. A. Pattern of disease recurrence and prognostic factors in patients with osteosarcoma treated with contemporary chemotherapy. Cancer 98, 2447–2456, 10.1002/cncr.11799 (2003). 14635080

[b11] Gajewski T. F., Schreiber H. & Fu Y. X. Innate and adaptive immune cells in the tumor microenvironment. Nat Immunol. 14, 1014–1022, 10.1038/ni.2703 (2013). 24048123 PMC4118725

[b12] Finn O. J. Immuno-oncology: understanding the function and dysfunction of the immune system in cancer. Ann Oncol. 23 Suppl 8, viii6-9, 10.1093/annonc/mds256 (2012). PMC4085883 22918931

[b13] Grivennikov S. I., Greten F. R. & Karin M. Immunity, inflammation, and cancer. Cell 140, 883–899, 10.1016/j.cell.2010.01.025 (2010). 20303878 PMC2866629

[b14] Schreiber R. D., Old L. J. & Smyth M. J. Cancer immunoediting: integrating immunity’s roles in cancer suppression and promotion. Science 331, 1565–1570, 10.1126/science.1203486 (2011). 21436444

[b15] Champiat S., Ferte C., Lebel-Binay S., Eggermont A. & Soria J. C. Exomics and immunogenics: Bridging mutational load and immune checkpoints efficacy. Oncoimmunology 3, e27817, 10.4161/onci.27817 (2014). 24605269 PMC3937193

[b16] Broadhead M. L., Clark J. C., Myers D. E., Dass C. R. & Choong P. F. The molecular pathogenesis of osteosarcoma: a review. Sarcoma 2011, 959248, 10.1155/2011/959248 (2011). 21559216 PMC3087974

[b17] Gorlick R. Current concepts on the molecular biology of osteosarcoma. Cancer Treat Res. 152, 467–478, 10.1007/978-1-4419-0284-9_27 (2009). 20213409

[b18] Kansara M., Teng M. W., Smyth M. J. & Thomas D. M. Translational biology of osteosarcoma. Nat Rev Cancer 14, 722–735, 10.1038/nrc3838 (2014). 25319867

[b19] Jones M. J. & Jallepalli P. V. Chromothripsis: chromosomes in crisis. Dev Cell 23, 908–917, 10.1016/j.devcel.2012.10.010 (2012). 23153487 PMC3514072

[b20] Stephens P. J. . Massive genomic rearrangement acquired in a single catastrophic event during cancer development. Cell 144, 27–40, 10.1016/j.cell.2010.11.055 (2011). 21215367 PMC3065307

[b21] Chen X. . Recurrent somatic structural variations contribute to tumorigenesis in pediatric osteosarcoma. Cell Rep. 7, 104–112, 10.1016/j.celrep.2014.03.003 (2014). 24703847 PMC4096827

[b22] Lussier D. M. . Enhanced T-cell immunity to osteosarcoma through antibody blockade of PD-1/PD-L1 interactions. J Immunother 38, 96–106, 10.1097/CJI.0000000000000065 (2015). 25751499 PMC6426450

[b23] Shen J. K. . Programmed cell death ligand 1 expression in osteosarcoma. Cancer Immunol Res. 2, 690–698, 10.1158/2326-6066.CIR-13-0224 (2014). 24866169 PMC4082476

[b24] Carreno B. M. & Collins M. The B7 family of ligands and its receptors: new pathways for costimulation and inhibition of immune responses. Annu Rev Immunol. 20, 29–53, 10.1146/annurev.immunol.20.091101.091806 (2002). 11861596

[b25] Dong H. & Chen L. B7-H1 pathway and its role in the evasion of tumor immunity. J Mol Med (Berl) 81, 281–287, 10.1007/s00109-003-0430-2 (2003). 12721664

[b26] Keir M. E., Butte M. J., Freeman G. J. & Sharpe A. H. PD-1 and its ligands in tolerance and immunity. Annu Rev Immunol. 26, 677–704, 10.1146/annurev.immunol.26.021607.090331 (2008). 18173375 PMC10637733

[b27] Ansell S. M. . PD-1 blockade with nivolumab in relapsed or refractory Hodgkin’s lymphoma. N Engl J Med. 372, 311–319, 10.1056/NEJMoa1411087 (2015). 25482239 PMC4348009

[b28] Garon E. B. . Pembrolizumab for the treatment of non-small-cell lung cancer. N Engl J Med 372, 2018–2028, 10.1056/NEJMoa1501824 (2015). 25891174

[b29] McDermott D. F. & Atkins M. B. PD-1 as a potential target in cancer therapy. Cancer Med. 2, 662–673, 10.1002/cam4.106 (2013). 24403232 PMC3892798

[b30] Mullard A. New checkpoint inhibitors ride the immunotherapy tsunami. Nat Rev Drug Discov. 12, 489–492, 10.1038/nrd4066 (2013). 23812256

[b31] Swaika A., Hammond W. A. & Joseph R. W. Current state of anti-PD-L1 and anti-PD-1 agents in cancer therapy. Mol Immunol. 67, 4–17, 10.1016/j.molimm.2015.02.009 (2015). 25749122

[b32] Afanasiev O. K. . Merkel polyomavirus-specific T cells fluctuate with merkel cell carcinoma burden and express therapeutically targetable PD-1 and Tim-3 exhaustion markers. Clin Cancer Res. 19, 5351–5360, 10.1158/1078-0432.CCR-13-0035 (2013). 23922299 PMC3790865

[b33] Chen B. J. . PD-L1 expression is characteristic of a subset of aggressive B-cell lymphomas and virus-associated malignancies. Clin Cancer Res. 19, 3462–3473, 10.1158/1078-0432.CCR-13-0855 (2013). 23674495 PMC4102335

[b34] Herbst R. S. . Predictive correlates of response to the anti-PD-L1 antibody MPDL3280A in cancer patients. Nature 515, 563–567, 10.1038/nature14011 (2014). 25428504 PMC4836193

[b35] Wimberly H. . PD-L1 Expression Correlates with Tumor-Infiltrating Lymphocytes and Response to Neoadjuvant Chemotherapy in Breast Cancer. Cancer Immunol Res. 3, 326–332, 10.1158/2326-6066.CIR-14-0133 (2015). 25527356 PMC4390454

[b36] Lussier D. M., Johnson J. L., Hingorani P. & Blattman J. N. Combination immunotherapy with alpha-CTLA-4 and alpha-PD-L1 antibody blockade prevents immune escape and leads to complete control of metastatic osteosarcoma. J Immunother Cancer 3, 21, 10.1186/s40425-015-0067-z (2015). 25992292 PMC4437699

[b37] Chen D. S., Irving B. A. & Hodi F. S. Molecular pathways: next-generation immunotherapy–inhibiting programmed death-ligand 1 and programmed death-1. Clin Cancer Res. 18, 6580–6587, 10.1158/1078-0432.CCR-12-1362 (2012). 23087408

[b38] Zhang Q. W. . Prognostic significance of tumor-associated macrophages in solid tumor: a meta-analysis of the literature. PLoS One 7, e50946, 10.1371/journal.pone.0050946 (2012). 23284651 PMC3532403

[b39] Gwak J. M., Jang M. H., Kim D. I., Seo A. N. & Park S. Y. Prognostic value of tumor-associated macrophages according to histologic locations and hormone receptor status in breast cancer. PLoS One 10, e0125728, 10.1371/journal.pone.0125728 (2015). 25884955 PMC4401667

[b40] Laoui D. . Tumor-associated macrophages in breast cancer: distinct subsets, distinct functions. Int J Dev Biol. 55, 861–867, 10.1387/ijdb.113371dl (2011). 22161841

[b41] Leek R. D. . Association of macrophage infiltration with angiogenesis and prognosis in invasive breast carcinoma. Cancer Res. 56, 4625–4629 (1996). 8840975

[b42] Quatromoni J. G. & Eruslanov E. Tumor-associated macrophages: function, phenotype, and link to prognosis in human lung cancer. Am J Transl Res. 4, 376–389 (2012). 23145206 PMC3493031

[b43] Jersmann H. P. Time to abandon dogma: CD14 is expressed by non-myeloid lineage cells. Immunol Cell Biol. 83, 462–467, 10.1111/j.1440-1711.2005.01370.x (2005). 16174094

[b44] Buddingh E. P. . Tumor-infiltrating macrophages are associated with metastasis suppression in high-grade osteosarcoma: a rationale for treatment with macrophage activating agents. Clin Cancer Res. 17, 2110–2119, 10.1158/1078-0432.CCR-10-2047 (2011). 21372215

[b45] Kleinerman E. S. . Phase II study of liposomal muramyl tripeptide in osteosarcoma: the cytokine cascade and monocyte activation following administration. J Clin Oncol. 10, 1310–1316 (1992). 1634921 10.1200/JCO.1992.10.8.1310

[b46] Pahl J. H. . Macrophages inhibit human osteosarcoma cell growth after activation with the bacterial cell wall derivative liposomal muramyl tripeptide in combination with interferon-gamma. J Exp Clin Cancer Res. 33, 27, 10.1186/1756-9966-33-27 (2014). 24612598 PMC4007518

[b47] Endo-Munoz L., Evdokiou A. & Saunders N. A. The role of osteoclasts and tumour-associated macrophages in osteosarcoma metastasis. Biochim Biophys Acta 1826, 434–442, 10.1016/j.bbcan.2012.07.003 (2012). 22846337

[b48] Endo-Munoz L. . Loss of osteoclasts contributes to development of osteosarcoma pulmonary metastases. Cancer Res. 70, 7063–7072, 10.1158/0008-5472.CAN-09-4291 (2010). 20823153

[b49] Avnet S. . Increased osteoclast activity is associated with aggressiveness of osteosarcoma. Int J Oncol. 33, 1231–1238 (2008). 19020756

[b50] Akiyama T. . Systemic RANK-Fc protein therapy is efficacious against primary osteosarcoma growth in a murine model via activity against osteoclasts. J Pharm Pharmacol. 62, 470–476, 10.1211/jpp/62.04.0009 (2010). 20604836

[b51] Fang X., Jiang C. & Xia Q. Effectiveness evaluation of dendritic cell immunotherapy for osteosarcoma on survival rate and *in vitro* immune response. Genet Mol Res. 14, 11763–11770, 10.4238/2015.October.2.10 (2015). 26436501

[b52] Himoudi N. . Lack of T-cell responses following autologous tumour lysate pulsed dendritic cell vaccination, in patients with relapsed osteosarcoma. Clin Transl Oncol. 14, 271–279, 10.1007/s12094-012-0795-1 (2012). 22484634

[b53] Kawano M., Nishida H., Nakamoto Y., Tsumura H. & Tsuchiya H. Cryoimmunologic antitumor effects enhanced by dendritic cells in osteosarcoma. Clin Orthop Relat Res. 468, 1373–1383, 10.1007/s11999-010-1302-z (2010). 20232181 PMC2853649

[b54] Kawano M. . Dendritic cells combined with anti-GITR antibody produce antitumor effects in osteosarcoma. Oncol Rep. 34, 1995–2001, 10.3892/or.2015.4161 (2015). 26239052

[b55] Muraro M. . Interactions between osteosarcoma cell lines and dendritic cells immune function: An *in vitro* study. Cell Immunol. 253, 71–80, 10.1016/j.cellimm.2008.05.002 (2008). 18565501

[b56] Edwards J. R. . Lymphatics and bone. Hum Pathol. 39, 49–55, 10.1016/j.humpath.2007.04.022 (2008). 17904616

[b57] Bacci G. . Neoadjuvant chemotherapy for osteosarcoma of the extremities with metastases at presentation: recent experience at the Rizzoli Institute in 57 patients treated with cisplatin, doxorubicin, and a high dose of methotrexate and ifosfamide. Ann Oncol. 14, 1126–1134 (2003). 12853357 10.1093/annonc/mdg286

[b58] Hattori H. & Yamamoto K. Lymph node metastasis of osteosarcoma. J Clin Oncol 30, e345–e349, 10.1200/JCO.2012.42.3384 (2012). 23032623

[b59] Sowers R. . Impairment of Methotrexate Transport Is Common in Osteosarcoma Tumor Samples. Sarcoma 2011, 834170, 10.1155/2011/834170 (2011). 21234348 PMC3017953

[b60] Abdeen A. . Correlation between clinical outcome and growth factor pathway expression in osteogenic sarcoma. Cancer 115, 5243–5250, 10.1002/cncr.24562 (2009). 19670450 PMC7251638

[b61] Osborne T. S. . Evaluation of eIF4E Expression in an Osteosarcoma Specific Tissue Microarray. Journal of pediatric hematology/oncology 33, 524–528, 10.1097/MPH.0b013e318223d0c1 (2011). 21941146 PMC3179611

[b62] Roth M. . Targeting Glycoprotein NMB With Antibody-Drug Conjugate, Glembatumumab Vedotin, for the Treatment of Osteosarcoma. Pediatr Blood Cancer 63, 32–38, 10.1002/pbc.25688 (2016). 26305408

[b63] Kubo T. . Platelet-derived growth factor receptor as a prognostic marker and a therapeutic target for imatinib mesylate therapy in osteosarcoma. Cancer 112, 2119–2129, 10.1002/cncr.23437 (2008). 18338812

