# Supplementary material for: Immune infiltration and PD-L1 expression in the tumor microenvironment are prognostic in osteosarcoma
Source: Sci Rep. 2016 Jul 26;6:30093. doi: 10.1038/srep30093 (PMC4960483; doi:10.1038/srep30093)
Supplement: Supplementary Information [file srep30093-s1.doc]

**Immune infiltration and PD-L1 expression in the tumor microenvironment are prognostic in osteosarcoma**

Pratistha Koirala^1^, Michael E. Roth^2^, Jonathan Gill^2^, Sajida Piperdi^2^, Jordan M. Chinai^3^, David S. Geller^2, 4^, Bang H. Hoang^4^, Amy Park^2^, Michael A. Fremed^1, 5^, Xingxing Zang^3^, Richard Gorlick^1, 2^

**Supplementary Table 1,** Patient demographic information for the tumor microarray specimens.

|  | **Biopsy** | **Definitive Surgery** | **Metastatic Tissue** |
| --- | --- | --- | --- |
|  | **(n=13)** | **(n=28)** | **(n=13)** |
|  |  |  |  |
| **Median age years, (SD)** | 16 (22) | 20 (20) | 16 (4) |
| **Huvos Response** |  |  |  |
| **Good** | 6 | 15 | 1 |
| **Poor** | 6 | 6 | 7 |
| **Metastasis** |  |  |  |
| **Present** | 7 | 12 | 13 |
| **Absent** | 6 | 15 | 0 |

**Supplementary Table 2, PD-L1 and TIL/APC co-expression.**

|  | **PD-L1 positive** | **PD-L1 negative** | **p-value** |
| --- | --- | --- | --- |
| **PD-1 positive** | 92% | 36% | p=0.002 |
| **CD3 positive** | 92% | 56% | p=0.04 |
| **CD1a positive** | 83% | 31% | p=0.002 |
| **CD56 positive** | 100% | 67% | p=0.02 |
| **CD68 positive** | 92% | 50% | p=0.16 |
| **CD8 positive** | 75% | 47% | p=0.18 |
| **CD20 positive** | 67% | 47% | p=0.32 |

PD-L1 expression is significantly associated with tumor infiltration by multiple immune cell subtypes.

**Supplementary Table 3**, Primary and secondary antibody list.

| **Antibody** | **Dilution (1/n)** | **Species** | **Positive control** | **Clone** | **Catalog Number** |
| --- | --- | --- | --- | --- | --- |
| **CD3** | 50 | Rabbit | Spleen | SP7 | M3074 |
| **CD4** | 50 | Mouse | Spleen | SP35 | M3354 |
| **CD8** | 100 | Rabbit | Spleen | SP16 | M3162 |
| **CD20** | 200 | Rabbit | Spleen | N/A | E2560 |
| **CD68** | 50 | Mouse | Skin | PG-M1 | MS1808 |
| **CD56** | 50 | Mouse | Brain | 123C3 | MA5-16445 |
| **CD1a** | 50 | Mouse | Skin | O10 | MS1856 |
| **PD-1** | 50 | Mouse | Tonsil | NAT105 | Ab52587 |
| **PD-L1** | 50 | Rabbit | Placenta | SP142 | M4424 |
| **Anti-mouse** | 200 | Goat | N/A | 1613 | SC-2039 |
| **Anti-rabbit** | 200 | Donkey | N/A | F2206 | SC-2089 |

**Supplementary Figure 1**


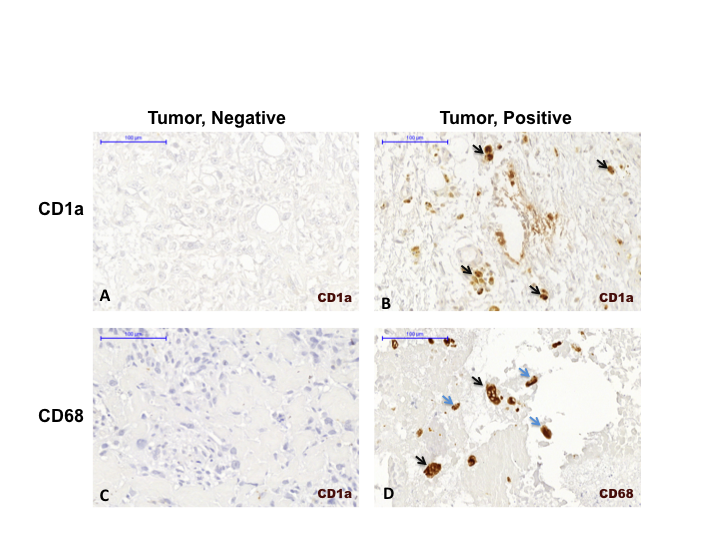


**Figure S1**. CD1a Dendritic Cells and CD68 positive Macrophages/Osteoclasts are present in osteosarcoma. **A,** osteosarcoma tumor lacking infiltration by CD1a dendritic cells. **B,** osteosarcoma tumor that is infiltrated by multiple dendritic cells. **C,** osteosarcoma tumor lacking infiltration by CD68 positive macrophages or osteoclasts **D.** osteosarcoma tumor containing both osteoclasts (blue arrows) and infiltrating macrophages (black arrows), both of which are CD68 positive. Scale bar represents 100 μm.

**Supplementary Figure 2**

**
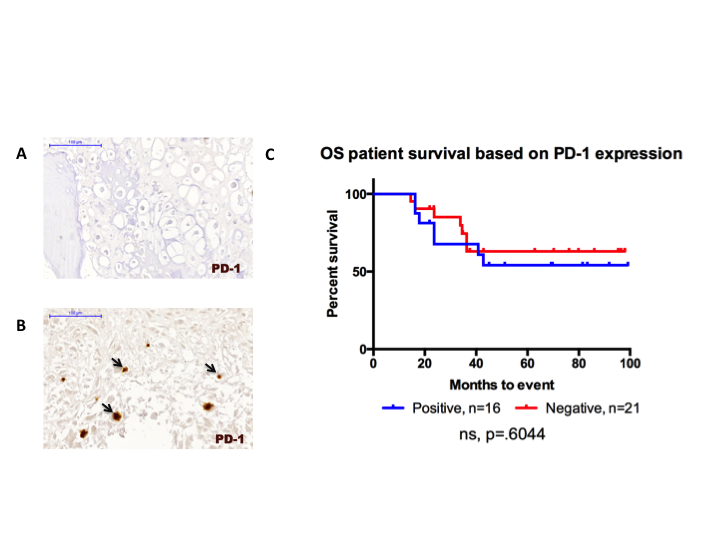
**

**Figure S2**. PD-1 is expressed in osteosarcoma cells. **A-B,** PD-1 is expressed in a subset of osteosarcoma primary tumor. **C**, PD-1 expression is not significantly associated with differential EFS. Scale bar represents 100 μm.

**Supplementary Figure 3**

**
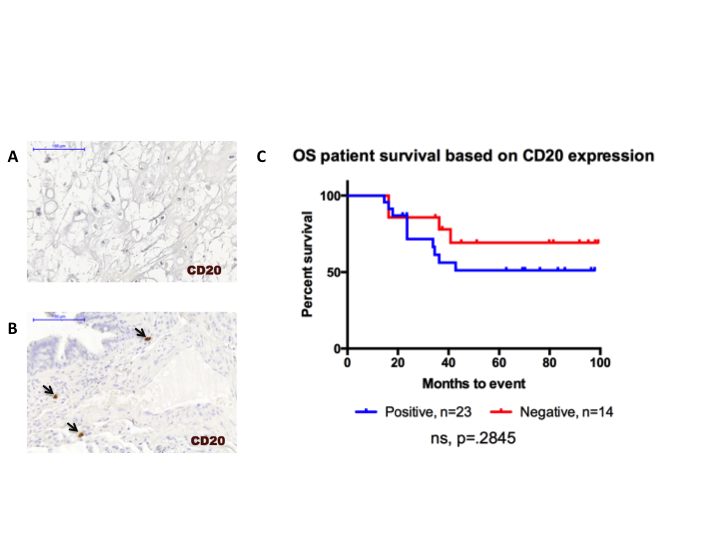
**

**Figure S3**. CD20 positive B cells are present in osteosarcoma cells. **A,** osteosarcoma tumor lacking infiltration by CD20 positive B cells**. B,** osteosarcoma tumor that is infiltrated by multiple CD20 positive B cells. **C**, CD20 expression is not significantly associated with differential EFS. Scale bar represents 100 μm.

**Supplementary Figure 4**

**
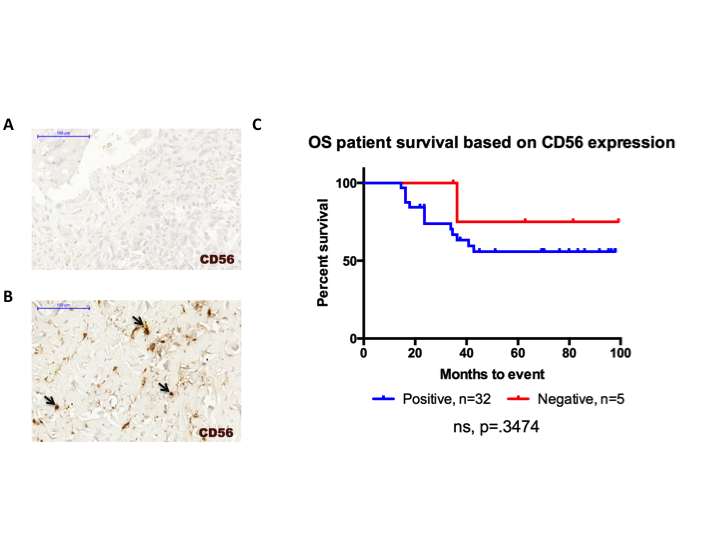
**

**Figure S4**. CD56 positive NK cells are present in osteosarcoma cells. **A,** osteosarcoma tumor lacking infiltration by CD56 positive NK cells**. B,** osteosarcoma tumor that is infiltrated by multiple CD56 positive NK cells. **C**, CD56 expression is not significantly associated with differential EFS. Scale bar represents 100 μm.

**Supplementary Figure 5**

**
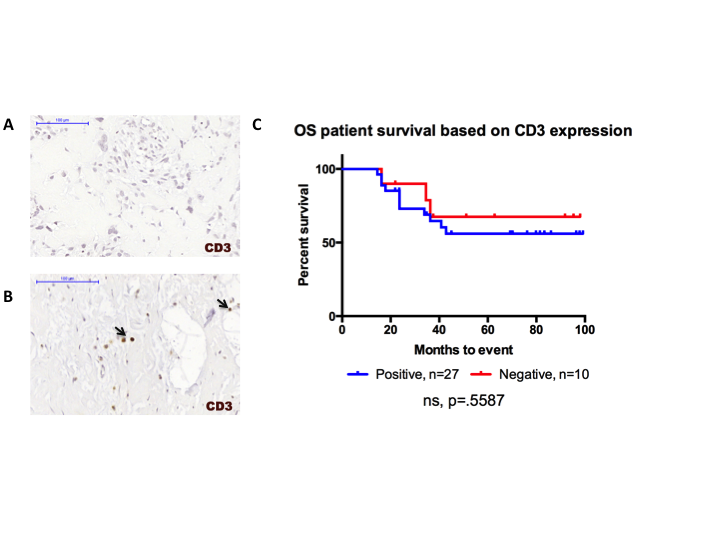
**

**Figure S5**. CD3 positive T cells are present in osteosarcoma cells. **A,** osteosarcoma tumor lacking infiltration by CD3 positive T cells**. B,** osteosarcoma tumor that is infiltrated by multiple CD3 positive T cells. **C**, CD3 expression is not significantly associated with differential EFS. Scale bar represents 100 μm.

**Supplementary Figure 6**


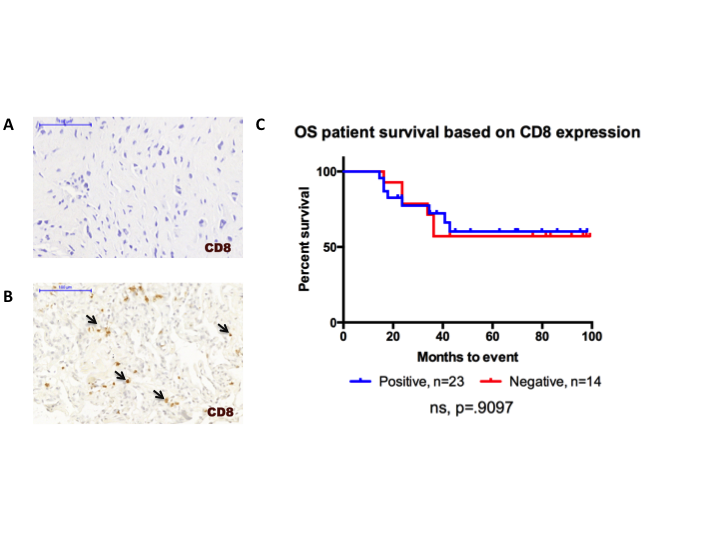


**Figure S6**. CD8 positive Cytotoxic T cells are present in osteosarcoma cells. **A,** Osteosarcoma tumor lacking infiltration by CD8 positive T cells**. B,** osteosarcoma tumor that is infiltrated by multiple CD8 positive T cells. **C**, CD8 expression is not significantly associated with differential EFS. Scale bar represents 100 μm.
